# An interpretable multiparametric radiomics model for the diagnosis of schizophrenia using magnetic resonance imaging of the corpus callosum

**DOI:** 10.1038/s41398-021-01586-2

**Published:** 2021-09-06

**Authors:** Minji Bang, Jihwan Eom, Chansik An, Sooyon Kim, Yae Won Park, Sung Soo Ahn, Jinna Kim, Seung-Koo Lee, Sang-Hyuk Lee

**Affiliations:** 1 Department of Psychiatry, CHA Bundang Medical Center, CHA University School of Medicine, Seongnam, Republic of Korea; 2 Department of Computer Science, Yonsei University, Seoul, Republic of Korea; 3 Research and Analysis Team, National Health Insurance Service Ilsan Hospital, Goyang, Republic of Korea; 4 Department of Statistics and Data Science, Yonsei University, Seoul, Republic of Korea; 5 Department of Radiology and Research Institute of Radiological Science and Center for Clinical Imaging Data Science, Yonsei University College of Medicine, Seoul, Republic of Korea

**Keywords:** Diagnostic markers, Schizophrenia

## Abstract

There is a growing need to develop novel strategies for the diagnosis of schizophrenia using neuroimaging biomarkers. We investigated the robustness of the diagnostic model for schizophrenia using radiomic features from T1-weighted and diffusion tensor images of the corpus callosum (CC). A total of 165 participants [86 schizophrenia and 79 healthy controls (HCs)] were allocated to training (*N* = 115) and test (*N* = 50) sets. Radiomic features of the CC subregions were extracted from T1-weighted, apparent diffusion coefficient (ADC), and fractional anisotropy (FA) images (*N* = 1605). Following feature selection, various combinations of classifiers were trained, and Bayesian optimization was adopted in the best performing classifier. Discrimination, calibration, and clinical utility of the model were assessed. An online calculator was constructed to offer the probability of having schizophrenia. SHapley Additive exPlanations (SHAP) was applied to explore the interpretability of the model. We identified 30 radiomic features to differentiate participants with schizophrenia from HCs. The Bayesian optimized model achieved the highest performance, with an area under the curve (AUC), accuracy, sensitivity, and specificity of 0.89 (95% confidence interval: 0.81–0.98), 80.0, 83.3, and 76.9%, respectively, in the test set. The final model offers clinical probability in an online calculator. The model explanation by SHAP suggested that second-order features from the posterior CC were highly associated with the risk of schizophrenia. The multiparametric radiomics model focusing on the CC shows its robustness for the diagnosis of schizophrenia. Radiomic features could be a potential source of biomarkers that support the biomarker-based diagnosis of schizophrenia and improve the understanding of its neurobiology.

## Introduction

Schizophrenia is a highly disabling psychiatric disorder with an unclear etiology and pathogenesis. A large body of evidence indicates that a constellation of cognitive, perceptual, and affective disturbances in schizophrenia originates from aberrations in neural connectivity among distinct brain regions [1]. However, despite the current notion of schizophrenia as a disorder of brain dysconnectivity, its diagnosis is exclusively based on a clinical description of the observable features of psychopathology. Therefore, it is crucial to develop novel strategies for the precise diagnosis of schizophrenia using specific and reliable biomarkers of brain connectivity.

The corpus callosum (CC) is the largest white matter structure in the brain, responsible for transferring and integrating a vast amount of multimodal information across the two hemispheres [2]. Neuroimaging abnormalities in the CC have been consistently found in patients with schizophrenia, suggesting the involvement of aberrant interhemispheric communication via the CC in the pathogenesis of schizophrenia. Structural magnetic resonance imaging (MRI) studies have reported morphological alterations in callosal shape [3, 4] and smaller size of the CC and its subregions [5, 6] in patients with schizophrenia compared to that of healthy controls (HCs). Diffusion tensor imaging (DTI), which allows for the in vivo assessment of microstructural features of the white matter, revealed myelin and axonal alterations of the CC in patients with schizophrenia, which cannot be captured by standard MRI [7]. The latest large-scale collaborative study on white matter abnormalities in schizophrenia showed that a lower fractional anisotropy (FA) and higher apparent diffusion coefficient (ADC) in the CC are among the most reliable findings with a large effect size that differentiated patients with schizophrenia from HCs [8]. However, none of the neuroimaging measures of the CC are diagnostic of schizophrenia because similar alterations are found in various psychiatric disorders, including autism [9, 10] and bipolar disorder [11]. Furthermore, single-value parameters derived from neuroimaging data, which measure the volume, area, or fiber integrity, do not fully capture the subtle and complex neuropathological changes in the CC, underlying the development of schizophrenia. The pathogenesis of schizophrenia involves microscopic alterations in tissue characteristics of the brain, resulting from multiple genetic, molecular, and cytoarchitectural factors. Therefore, an approach that quantifies the spatial distribution of microscopic tissue heterogeneity, which cannot be assessed with conventional imaging parameters, would be a promising alternative to improve the diagnosis of schizophrenia on the basis of neurobiology.

Radiomics is a new, advanced analytic technique that quantifies and extracts high-dimensional imaging features to aid clinical decision-making using medical image-based biomarkers [12]. Radiomics aims to discover meaningful “hidden” information within radiological images, which is inaccessible with single-value approaches. Radiomics relies on computational techniques to translate radiologic images into quantitative image descriptors pertaining to the shape and texture information of a region of interest (ROI) [13]. Radiomic features include shape, first-order features, and second-order features. Shape descriptors are calculated based on the two- and three-dimensional size and shape of the ROI. On the other hand, first-order and second-order features are calculated based on signal intensity values within the ROI by mathematical equations. First-order features reflect the intensity distribution of an ROI, while second-order features reflect spatial heterogeneity [14]. Radiomics has a wide potential as a diagnostic and prognostic biomarker in brain tumors [15] as well as neurodegenerative diseases, such as Alzheimer’s and Parkinson’s disease [16, 17]. Recent studies on schizophrenia have also shown promising results in differentiating patients with schizophrenia from HCs using a radiomics model with structural MRI of the hippocampal subfields [18] and resting-state functional connectivity [19] and in predicting short-term treatment responses after electroconvulsive therapy in patients with treatment-resistant schizophrenia, using radiomic features extracted from structural and diffusion MRI [20]. The clinical utility of these radiomics models built through machine learning may be limited due to the lack of interpretability of the “black-box” system. However, this problem could be addressed using explanatory techniques, such as SHapley Additive exPlanations (SHAP) that estimate a value of importance for each radiomic feature in the built model and facilitate informed clinical decision-making [21].

To the best of our knowledge, there is no study to date that tests a potential diagnostic model of schizophrenia using radiomics based on multiparametric DTI and T1-weighted images (T1) focusing on the interhemispheric connectivity of the brain. Herein, we aimed to investigate the ability of a multiparametric radiomics model to diagnose schizophrenia using MRI of the CC. We hypothesized that radiomic analysis of the CC would reveal distinct combinations of neuroimaging features reflecting subtle and complex alterations in tissue characteristics of the CC, which would differentiate patients with schizophrenia from HCs. Furthermore, the interpretability of the diagnostic prediction made by the multiparametric radiomics model was explored by the application of SHAP.

## Materials and methods

### Participants

The participants with schizophrenia were recruited from among those receiving psychiatric treatment at the Department of Psychiatry, CHA Bundang Medical Center (Seongnam, Republic of Korea). They were diagnosed with schizophrenia based on the DSM-IV-TR or DSM-5 criteria [22, 23]. We only included patients who had no history of other psychiatric disorders and were antipsychotic-naïve or free of antipsychotics for at least 6 months. HCs were recruited from the local community through online and print advertisements, and we ensured that they had no personal or first-relative family history of psychiatric disorders. The exclusion criteria were (1) a current or past history of neurological or neurodevelopmental disorders, substance-related problems, or head trauma with loss of consciousness, (2) left-handedness, and (3) any other contraindications for undergoing MRI. A total of 165 participants (86 with schizophrenia and 79 HCs) were finally enrolled in this study. The study participants of both groups were allocated to training (*N* = 115; 60 with schizophrenia and 55 HCs) and test (*N* = 50; 26 with schizophrenia and 24 HCs) sets (Table 1).

**Table 1:** Demographic and clinical profiles of the study participants in the training and test sets.

	Training set (*N* = 115)		Test set (*N* = 50)	
	Schizophrenia (*N* = 60)	HCs (*N* = 55)	Schizophrenia (*N* = 26)	HCs (*N* = 24)
Age (y)	34.3 (9.5)	37.1 (8.3)	37.0 (11.2)	40.6 (12.4)
Sex				
Male	22 (36.7%)	25 (45.5%)	9 (34.6%)	7 (29.2%)
Female	38 (63.3%)	30 (54.5%)	17 (65.4%)	17 (70.8%)
Number of episodes				
First episode	49 (81.7%)		17 (65.4%)	
Second episode	6 (10.0%)		4 (15.4%)	
Third episodes	5 (8.3%)		5 (19.2%)	
Duration of illness (mo)	15.6 (23.7)		25.0 (33.7)	
Antipsychotic exposure				
Naïve	53 (88.3%)		23 (88.5%)	
>6 months free	7 (11.7%)		3 (11.5%)	
Duration of antipsychotics before MRI scan (d)	6.2 (6.0)		4.7 (4.9)	
Dose of antipsychotics at MRI scan (mg/d)^a^	504.3 (301.8)		429.5 (220.7)	
PANSS				
Positive symptoms	29.5 (7.7)		30.4 (5.6)	
Negative symptoms	20.9 (7.0)		20.4 (6.3)	
General psychopathology	59.6 (14.2)		57.8 (13.5)	

There were no significant differences between groups and between sets on any variable.

*HCs* healthy controls, *MRI* magnetic resonance imaging, *PANSS* positive and negative syndrome scale.

^a^All participants with schizophrenia were taking atypical antipsychotics and chlorpromazine equivalent doses were calculated.

The study protocol was reviewed and approved by the Institutional Review Board of CHA Bundang Medical Center, in accordance with the latest version of the Declaration of Helsinki and principles of Good Clinical Practice. Written informed consent was obtained from all study participants after the study procedures had been fully explained.

### MRI data acquisition and preprocessing

T1 and diffusion-weighted images were acquired using a 3.0-Tesla scanner (GE Signa HDxt; GE Healthcare, Milwaukee, WI, USA) equipped with an eight-channel phased-array head coil, at CHA Bundang Medical Center (Seongnam, Republic of Korea). Detailed information on the MRI protocol is presented in Supplementary Material S1.

The anatomical segmentation of the CC was performed on T1 using the standard “recon-all” pipeline implemented in the FreeSurfer software (version 7.1.0; http://freesurfer.net). The CC was automatically segmented into five subregions: anterior, mid-anterior, central, mid-posterior, and posterior. Each of the subregional masks was extracted from individual T1. The preprocessing of DTI data was performed using the Functional MRI of the Brain’s (FMRIB’s) Diffusion Toolbox, which is a part of FMRIB’s Software Library (version 6.0; https://fsl.fmrib.ox.ac.uk/fsl/). The DT images underwent standard preprocessing steps, including brain extraction and correction for head motion and eddy-current distortion. ADC and FA maps were created by fitting a tensor model to the corrected diffusion data. After image resampling to 1-mm isovoxels, the nonuniformity of low-frequency intensity was corrected with the N4 bias field correction algorithm. T1 was co-registered to ADC and FA maps by the affine transformation with normalized mutual information as a cost function.

### Radiomic feature extraction

**Fig. 1:**
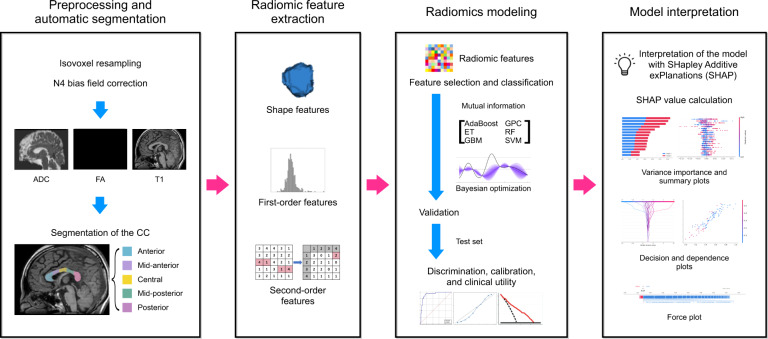
Workflow of image preprocessing, radiomics feature extraction, and machine learning. ADC apparent diffusion coefficient, FA fractional anisotropy, T1 T1-weighted image, CC corpus callosum, ET extra trees, GBM gradient boosting machine, GPC Gaussian process classification, RF random forest, SVM support vector machine.

Figure 1 shows the radiomics pipeline of the study. Radiomic feature extraction from the CC subregions was performed using py-Radiomics (version 2.0; http://www.radiomics.io/pyradiomics.html) [24], which conformed to the Image Biomarker Standardization Initiative [25]. The radiomic features included 14 shape features, 18 first-order features, and 75 second-order features [such as gray-level co-occurrence matrix (GLCM; *N* = 24), gray-level run-length matrix (GLRLM; *N* = 16), gray-level size zone matrix (*N* = 16), gray-level dependence matrix (*N* = 14), and neighboring gray tone difference matrix (*N* = 5); Supplementary Material S3]. A total of 1605 (107 features × 5 subregions of the CC × 3 sequences) radiomic features were extracted (Supplementary Material S2).

### Construction of the radiomics model for the assessment of discrimination, calibration, and clinical utility

The feature selection and machine learning processes were performed using Python 3 with the Scikit-Learn library module (version 0.21.2). All radiomic features were Min-Max normalized. Subsequently, feature selection was performed to reduce the number of highly correlated features and avoid overfitting by applying mutual information, which measures the mutual dependence between variables. The base radiomic classifiers were constructed using classifier models, such as AdaBoost, Extra Trees, gradient boosting machine, Gaussian process classification, random forest, and support vector machine, with tenfold cross-validation. To overcome data imbalance, each machine learning model was either trained without oversampling or with random oversampling examples (ROSE) technique (Supplementary Material S3). Thus, a total of 12 combinations with different oversampling techniques were trained and validated.

The discrimination performance was evaluated in the training set and validated in the test set. The predictive performance was quantified by calculating the AUC, accuracy, sensitivity, and specificity. To improve the predictive performance and avoid potential overfitting, Bayesian optimization was applied in the best performing base classifier, which searches the hyperparameter space for optimal combinations of the hyperparameters (Supplementary Material S4).

Discrimination and calibration are two different aspects of the performance of diagnostic models. Because good discrimination does not always ensure good calibration, and vice versa, calibration performance was also assessed based on the agreement between predicted and actual rates of schizophrenia in the calibration plot [26]. Calibration plots were created to determine whether the predicted probability correlated with the actual probabilities. The Brier score, which is the mean squared prediction error, was calculated to evaluate the overall performance of the radiomics model [27]. A Brier score close to 0 indicates a better calibration and discrimination performance of the model.

Decision curve analysis (DCA) was performed by calculating clinical “net benefits” at a range of threshold probabilities to validate the clinical utility of the radiomics model (Supplementary Material S5) [28]. Net benefit differs from accuracy metrics, such as discrimination and calibration, in that it incorporates the consequences of decisions made by a model or test [29].

### Model interpretability with SHAP

To interpret and understand the radiomic features from the radiomics model, SHAP was applied, which is a game-theoretic approach to explain the output of a tree-based machine learning model [21]. SHAP measures the contribution of each feature of a model to the increase or decrease of the probability of single output (i.e., the probability of the diagnosis of schizophrenia in this study; Supplementary Material S6).

### Statistical analysis

Demographic and clinical characteristics between participants with schizophrenia and HCs and between the training and test sets were compared using the independent *t*-test for continuous variables and the chi-squared test for categorical variables. The diagnostic performance of the radiomics model was compared with a logistic regression model with mean ADC and FA values from the CC subregions using AUC. Statistical procedures were conducted using the Statistical Package for the Social Sciences, version 26 (IBM Corp., Armonk, NY, USA). Statistical significance was set at *p* < 0.05.

## Results

### Discrimination, calibration, and clinical utility of the radiomics model

A total of 30 radiomic features (ten from anterior, three from mid-anterior, three from central, seven from mid-posterior, and seven from posterior subregions of the CC; 16 from ADC, five from FA, and nine from T1) were identified to differentiate participants with schizophrenia from HCs in the best performing classifier (Table 2). Supplementary Fig. 1 shows the heatmap of significant radiomic features.

**Table 2:** List of significant radiomic features to differentiate participants with schizophrenia from HCs.

Subregion	Image	Feature category	Feature name
Anterior	ADC	First-order (7)	10 percentile
			90 percentile
			Maximum
			Mean
			Median
			Root mean squared
			Variance
	FA	GLDM (1)	Large dependence emphasis
	T1	GLRLM (1)	Gray-level nonuniformity
		GLSZM (1)	Size zone nonuniformity
Mid-anterior	ADC	First-order (3)	90 percentile
			Minimum
			Interquartile range
Central	ADC	First-order (1)	90 percentile
	FA	NGTDM (1)	Complexity
		GLSZM (1)	Zone variance
Mid-posterior	ADC	First-order (1)	Minimum
	FA	First-order (2)	Mean
			Root mean squared
	T1	First-order (3)	Mean absolute deviation
			Minimum
			Robust mean absolute deviation
		GLRLM (1)	Gray-level nonuniformity normalized
Posterior	ADC	First-order (2)	Range
			Variance
		GLCM (1)	Correlation
			Information measure of correlation 1
	T1	First-order (2)	Mean absolute deviation
			Skewness
		GLRLM (1)	Run entropy

*HCs* healthy controls, *ADC* apparent diffusion coefficient, *FA* fractional anisotropy, *GLDM* gray-level dependence matrix, *GLRLM* gray-level run-length matrix, *GLSZM* gray-level size zone matrix, *NGTDM* neighboring gray tone difference matrix, *GLCM* gray-level co-occurrence matrix.

**Fig. 2:**
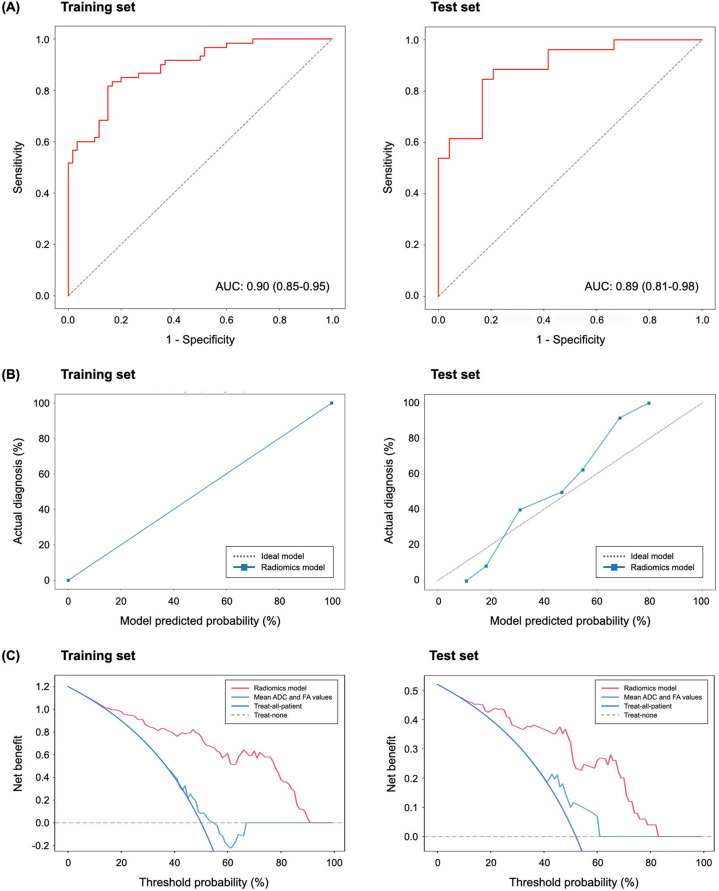
Discrimination, calibration, and clinical utility of the radiomics model for the diagnosis of schizophrenia in the training and test sets. **A** Receiver operating characteristics curves in the training and test sets. **B** Calibration plots of the radiomics model in the training and test sets. The x-axis represents the model predicted risk of schizophrenia. The y-axis represents the actual diagnosed proportion of schizophrenia. The gray line represents a perfect prediction by an ideal model, and the blue line represents the performance of the radiomics model. The closer fit between the diagonal dotted lines and solid lines shows a good prediction of schizophrenia. **C** Decision curves for the radiomics model in the training and test sets. The x-axis represents the threshold probability, which refers to a minimum probability of having a disease that patients require to justify treatment. The y-axis represents the net benefit, determined based on the difference between the expected benefit and harm associated with the diagnosis and treatment decision. The radiomics model shows superior net benefit in both the training and test sets within most of the range of threshold probability compared to either default strategies (treat-all or -none) or the model with mean ADC and FAs. AUC area under the curve, ADC apparent diffusion coefficient, FA fractional anisotropy.

The AUCs of the base classifiers ranged from 0.72 to 0.89 in the training set and 0.65 to 0.87 in the test set (Supplementary Fig. 2). The best performing base classifier in the training and test sets was achieved using the Extra Trees classifier model with ROSE technique; the AUC, accuracy, sensitivity, and specificity of the best performing base classifier in the test set were 0.87 [95% confidence interval (CI): 0.77–0.97], 78.0, 83.3, and 73.1%, respectively. After the Bayesian hyperparameter tuning, the optimized model with the best performing classifier (Extra Trees model with ROSE technique) achieved better predictive performance. In the Bayesian optimized model of the training set, the AUC, accuracy, sensitivity, and specificity were 0.90 (95% CI: 0.85–0.95), 81.7, 85.0, and 78.3%, respectively; in the test set, the AUC, accuracy, sensitivity, and specificity were 0.89 (95% CI: 0.81–0.98), 80.0, 83.3, and 76.9%, respectively (Fig. 2A). Supplementary Table 1 shows the performance of base classifiers and the optimized model in the test set.

The calibration plot showed that the predicted outcomes closely approximated the observed outcomes, indicating a good agreement between the predicted and actual rates of schizophrenia in the training and test sets (Fig. 2B). The Brier score was 0 and 0.16 in the training and test sets, respectively. The DCA result showed that the radiomics model added more net benefit compared to the default strategies of treating all or no subjects or the model with mean ADC and FA values when the threshold probability was within the range of 0–0.91 in the training set and 0–0.83 in the test set (Fig. 2C). Since the radiomics model had a higher net benefit than the strategy using mean ADC and FA values of the CC subregions within a reasonable range of threshold probability, it was considered clinically useful.

An online site has been made available to provide a free calculator based on the presented radiomics model (https://nhimc.shinyapps.io/sirano-schizoml/), which enables clinicians to access and estimate the probability of having schizophrenia. Representative results of the prediction of five participants (three patients with schizophrenia and two HCs) from the radiomics calculator are shown in Supplementary Fig. 3.

### Comparison of performance between the radiomics model and model with mean ADC and FA values

In the training set, the model with mean ADC and FA values from CC subregions showed an AUC, accuracy, sensitivity, and specificity of 0.63 (95% CI: 0.55–0.70), 60, 53.3, and 66.7%, respectively. In the test set, the model showed an AUC, accuracy, sensitivity, and specificity of 0.66 (95% CI: 0.50–0.82), 70.0, 54.2, and 84.6%, respectively. The radiomics model showed a significantly higher performance than the model with mean ADC and FA values from CC subregions, in both the training and test sets (*p* < 0.001 and *p* = 0.010, respectively).

### Model interpretability with SHAP

**Fig. 3:**
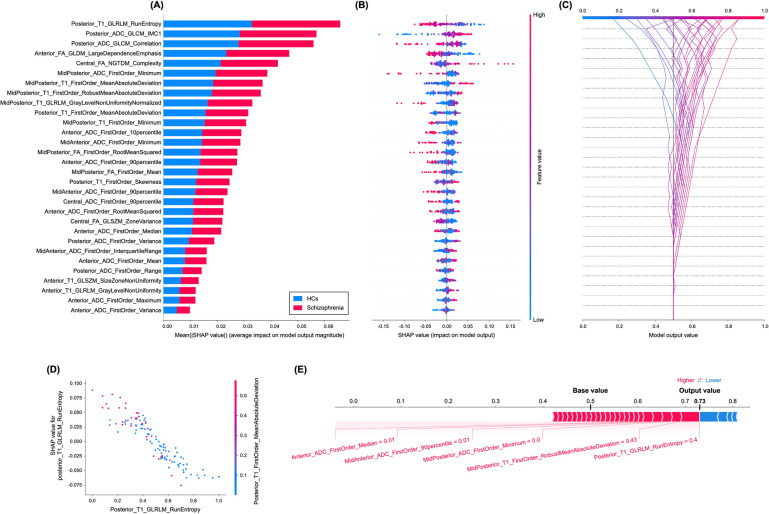
Model interpretability of the radiomics model for the diagnosis of schizophrenia with SHAP. **A** Variance importance plot listing the most significant variables in descending order. The top variables (such as posterior_T1_GLRLM_run entropy, posterior_ADC_GLCM_IMC1, and posterior_ADC_GLCM_correlation features) contribute more to the model than the bottom ones and thus have higher predictive power. **B** Summary plot of feature impact on the decision of the radiomics model and interaction between radiomics features in the model. A positive SHAP value indicates an increase in the risk of predicting schizophrenia and vice versa. The high value corresponds to a higher risk of schizophrenia. Each point corresponds to a prediction in each participant. **C** Decision plot showing how the radiomics model predicts schizophrenia. Moving from the bottom of the plot to the top, SHAP values for each feature are added to the model’s base value showing how each feature contributes to the overall prediction of schizophrenia. **D** An example of the dependence plot of the posterior_T1_GLRLM_run entropy feature, which is the top-most important feature. The plot shows how there is an approximately linear and negative trend between “posterior_T1_GLRLM_RunEntropy” and schizophrenia prediction, and that the “posterior_T1_GLRLM_RunEntropy” interacts with “posterior_T1_FirstOrder_MeanAbsoluteDeviation” frequently. **E** Force plot of a representative case of participants with schizophrenia from the test set, showing local interpretability. Note that the posterior_T1_GLRLM_RunEntropy largely pushes the model prediction score into the higher direction from the base value.

The SHAP values for each radiomic feature in the radiomics model were calculated. The variance importance plot, summary plot, decision plot, dependence plot, and force plot are shown in Fig. 3. For each prediction, a positive SHAP value indicates an increase in the risk of schizophrenia and vice versa. As observed in the plot, posterior_T1_GLRLM_run entropy, followed by posterior_ADC_GLCM_informational measure of correlation 1 (IMC1) and posterior_ADC_GLCM_correlation features, were the three most important risk factors.

## Discussion

In the present study, we assessed the diagnostic value of radiomic features extracted from multiparametric MRI of the CC to differentiate participants with schizophrenia from HCs. The radiomics model with Bayesian optimization, constructed with 30 significant features from the CC subregions, showed a robust performance with an AUC of 0.89 in the test set. Moreover, the model showed good calibration and had a greater net benefit for clinical decision-making in the diagnosis of schizophrenia. The finalized model was then implemented in an online interface. The robustness of the radiomics model was further supported by its significantly higher performance compared to the model with mean ADC and FA values from the CC subregions. The model explanation by SHAP suggested that radiomic features reflecting aberrations in tissue heterogeneity in the posterior CC were particularly associated with a higher risk of schizophrenia.

The neuropathology of schizophrenia involves alterations in the cytoarchitectural and synaptic organizations of the brain, including a loss and disarray of neurons. Previous postmortem studies found impaired myelinations of the CC and reduced numbers of lamina III pyramidal cells from which most CC axons originate in patients with schizophrenia [30, 31]. In vivo MRI data may contain biological information reflecting histological features of the CC in patients with schizophrenia, resulting in changes in signal intensity on DTI and T1. While T1 relaxation time is a direct reflection of tissue composition and cytoarchitectural organization [32], DTI represents white matter microstructure, including axonal coherence, fiber density, and myelin integrity [33]. The radiomics approach enables the comprehensive characterization of microscopic tissue heterogeneity and extraction of meaningful biomarkers from both modalities, providing different aspects of biological information. We found that selected radiomic features were distributed throughout the CC subregions, with distinct tissue characteristics in each of them. The majority of selected radiomic features were first-order features, followed by second-order features; none of the shape features, such as volume, area, and length, were included. This indicates that changes in interrelationships among voxel-based signal intensity as well as their distributions within the CC subregions, which are reflected in the first- and second-order features, could aid in differentiating participants with schizophrenia from HCs. Our findings of radiomic feature selection demonstrate the high potential of radiomics in capturing specific information regarding the neuropathological changes underlying the pathogenesis of schizophrenia, which cannot be quantified by a single parameter based on morphometry.

Radiomic analysis is based on modeling, and it offers an improved discriminatory power by utilizing supervised machine learning with binary classification. To establish the generalizability of the radiomics model, it is crucial to test its performance in a separate validation set [34]. Our radiomics model built with 30 selected radiomic features demonstrated its robustness by showing a significantly better performance compared to the model with mean ADC and FA values from the CC subregions, in the test set. Furthermore, the clinical usefulness of the radiomics model for the diagnosis of schizophrenia was demonstrated by the calibration plot and DCA. The calibration plot of the model showed a good agreement between the predicted and actual diagnoses of schizophrenia in the test set. The DCA result showed that the clinical application of the radiomics model for the diagnosis of schizophrenia is more beneficial than default strategies or the model using mean ADC and FA values within a reasonable range of threshold probability. Our result implies that the radiomics model has a substantial potential to support evidence-based decision-making in clinical practice by using objective and reliable neuroimaging biomarkers in the diagnosis of schizophrenia.

One major pitfall of the radiomics model is the lack of explainability in the “black-box” system, which limits its use in clinical practice. It is challenging for the clinicians to determine how the model arrives at a conclusion and to identify radiomic features that are critical in decision-making [35]. The advantage of SHAP is that it identifies certain patterns of complex machine learning algorithms and provides both global and local interpretability for the radiomics model [21]. We found that run entropy of GLRLM from T1 of the posterior CC was the most important factor in differentiating participants with schizophrenia from HCs, followed by IMC1 and correlation of GLCM from the ADC maps of the posterior CC. The GLRLM and GLCM are second-order radiomic features reflecting tissue heterogeneity by calculating the frequency of occurrence of pairs of voxels with specific values and a specified spatial relationship in an image [36]. The GLRLM describes heterogeneity in the distribution of run lengths, whereas GLCM describes heterogeneity in the distribution of co-occurring voxel values. Although the neurobiological underpinnings of radiomic features remain to be elucidated, microscopic aberrations in complex neuronal and glial organizations within the CC may alter the voxel signal intensities in DTI and T1 [37]. The posterior CC contains white matter tracts connecting the bilateral temporal, parietal, and occipital lobes, including multimodal sensory association cortices [38]. Our findings from SHAP imply that the disease-defining feature of schizophrenia may be associated with the “bottom-up” dysfunction in the integration of multisensory information, leading to cognitive and perceptual disturbances in patients with schizophrenia [39]. Future research is warranted to understand the neurobiological meaning of radiomic features in the pathogenesis of schizophrenia.

This study has a few limitations. First, this is a retrospective study from a single institution, and the sample size was relatively small. However, our study is the first to demonstrate the usefulness of the radiomics model with multiparametric MRI of the CC in diagnosing schizophrenia. Further studies with a larger dataset and external validation are needed. Second, although our participants with schizophrenia were initially antipsychotic-naïve or free of antipsychotics for at least 6 months, some MRI data were acquired after the administration of atypical antipsychotics. This may raise a concern regarding the confounding effect of antipsychotic medication. However, the influence of antipsychotics on the brain is controversial, and the mean duration of antipsychotic medication is very short in this study (5.7 ± 5.7 days). Third, the causality of changes in radiomic features of the CC subregions cannot be determined due to the cross-sectional nature of this study. However, given that the majority of participants with schizophrenia were in the early phase of their disorder, our findings may provide some clues to understand the early pathogenesis of schizophrenia.

In conclusion, the multiparametric radiomics model using selected features from the CC may offer a novel approach to improve the diagnosis of schizophrenia with good clinical applicability. Radiomic features extracted from neuroimaging data could be a potential source of biomarkers that support the objective diagnosis of schizophrenia and improve the understanding of its neurobiology. We expect that the estimation of microscopic tissue characteristics using radiomics and diagnostic modeling will promote the quality of clinical practice in managing patients with schizophrenia.

## Supplementary information

Supplementary Material

## Data Availability

The data supporting the findings of this study are not publicly available due to ethical restrictions for protecting participants’ confidentiality and privacy but are accessible from the corresponding author on reasonable request with the approval of the Institutional Review Board of CHA Bundang Medical Center.

## Code availability

The codes used in the radiomics analysis are made publicly available at https://github.com/AIM-Harvard/pyradiomics and https://github.com/scikit-learn/scikit-learn. The code used for SHapley Additive exPlanations (SHAP) analysis is available at https://github.com/slundberg/shap.
