# Supplementary material for: An interpretable multiparametric radiomics model for the diagnosis of schizophrenia using magnetic resonance imaging of the corpus callosum
Source: Transl Psychiatry. 2021 Sep 6;11:462. doi: 10.1038/s41398-021-01586-2 (PMC8421339; doi:10.1038/s41398-021-01586-2)
Supplement: Supplementary file 1 — Supplementary Material [file 41398_2021_1586_MOESM1_ESM.docx]

**Supplementary Material**

**S1. Magnetic resonance imaging (MRI) protocols**

MRI data acquisition was performed from all participants using a 3.0-Tesla scanner (GE Signa HDxt, GE Healthcare Milwaukee, WI, USA) equipped with an eight-channel phase array head coil at CHA Bundang Medical Center (Seongnam, Republic of Korea). High-resolution structural brain images were acquired with a T1-weighted three-dimensional fast spoiled gradient-recalled echo (FSPGR) sequence (repetition time [TR], 6.3 ms; echo time [TE], 2.1 ms; ﬂip angle, 12˚; ﬁeld of view [FOV], 256 × 256 mm^2^; matrix, 256 × 256; voxel size, 1 × 1 × 1 mm^3^). Diffusion-weighted images (DWI) were acquired with an echo planar imaging (EPI) sequence (TR, 17,000 ms; TE, 108 ms; FOV, 240 mm; matrix, 144 × 144; voxel size, 1.67 × 1.67 × 1.7 mm^3^). A double-echo option was applied to reduce eddy-current related distortions, and an 8-channel coil and ASSET (Array of Spatial Sensitivity Encoding Techniques; GE Healthcare) with a SENSE factor of two were used to reduce the impact of EPI spatial distortions. Seventy axial slices parallel to the anterior commissure-posterior commissure line were acquired in 51 directions with b = 900 s/mm^2^. Eight baseline scans with b = 0 s/mm^2^ were also acquired. The scanning time was 5 min for T1-weighted images and 17 min for DTIs.

**S2. Radiomic features**

Radiomic features included (1) 14 shape features, (2) 18 first-order features, and (3) 75 second-order features. Second features consist of gray level co-occurrence matrix (N = 24), gray level run-length matrix (N = 16), gray level size zone matrix (N = 16), gray level dependence matrix (N = 14), and neighboring gray tone difference matrix (N = 5). Detailed information on radiomics features is in the PyRadiomics 2.0 document (<http://www.radiomics.io/pyradiomics.html>).

***1. Shape features***

Shape features included descriptors of the three-dimensional size and shape of the region of interest (ROI). They are independent from the gray level intensity distribution in the ROI and were therefore only calculated on the non-derived image and mask.

Unless otherwise specified, features were derived from the approximated shape defined by the triangle mesh. To build this mesh, vertices (points) were first defined as points halfway on an edge between a voxel included in the ROI and one outside the ROI. By connecting these vertices, a mesh of connected triangles was obtained, with each triangle defined by three adjacent vertices, which shared each side with exactly one other triangle.

$N_{v}$ represents the number of voxels included in the ROI and $N_{f}$ represents the number of faces (triangles) defining the mesh. V is the volume of the mesh in mm^3^ and A is the surface area of the mesh in mm^2^.

| **Elongation** | $\sqrt{\frac{\lambda_{minor}}{\lambda_{major}}}$ | **Maximum 3D diameter** | The largest pairwise Euclidean distance between ROI surface mesh vertices |
| --- | --- | --- | --- |
| **Flatness** | $\sqrt{\frac{\lambda_{least}}{\lambda_{major}}}$ | **Mesh volume** | $V_{i}=\frac{O_{a_{i}}\cdot(O_{b_{i}}\times O_{c_{i}})}{6}$  $V=\sum_{i=1}^{N_{f}} V_{i}$ |
| **Least axis length** | $4\sqrt{\lambda_{least}}$ | **Minor axis length** | $4\sqrt{\lambda_{minor}}$ |
| **Major axis length** | 4$\sqrt{\lambda_{major}}$ | **Sphericity** | $\frac{\sqrt[3]{36\pi V^{2}}}{A}$ |
| **Maximum 2D diameter (column)** | The largest pairwise Euclidean distance between ROI surface mesh vertices in the row-column plane. | **Surface area** | $A_{i}=\frac{1}{2}\left\vert a_{i}b_{i}\times a_{i}c_{i} \right\vert$  $A=\sum_{i=1}^{N_{f}} A_{i}$ |
| **Maximum 2D diameter(row)** | The largest pairwise Euclidean distance between ROI surface mesh vertices in the column-slice plane. | **Surface area to volume ratio** | $\frac{A}{V}$ |
| **Maximum 2D diameter(slice)** | The largest pairwise Euclidean distance between ROI surface mesh vertices in the row-slice plane. | **Voxel volume** | $V_{voxel}=\sum_{k=1}^{N_{v}} V_{k}$ |

***2. First-order features***

First-order features describe the distribution of voxel intensities within the image region defined by the mask through commonly used and basic metrics.

X is a set of 𝑁𝑝 voxels included in the ROI. P(i) refers to the first-order histogram with 𝑁_𝑔_ discrete intensity levels, where 𝑁_𝑔_ is the number of non-zero bins, equally spaced from 0 with a width defined in the binWidth parameter and p(i) is the normalized first order histogram and equal to $\frac{P(i)}{N_{p}}$. 𝜖 is an arbitrarily small positive number (≈ 2.2 × 10^−16^).

| **Energy** | $\sum_{i=1}^{N_{p}} {(X\left( i \right)+c)}^{2}$ | **Interquartile Range** | $P_{75}{- P}_{25}$ |
| --- | --- | --- | --- |
| **Total energy** | $V_{voxel}\sum_{i=1}^{N_{p}} {(X\left( i \right)+c)}^{2}$ | **Range** | max(X)$-$ min(X) |
| **Entropy** | $-\sum_{i=1}^{N_{g}} p(i)\log_{2} (p\left( i \right)+ \epsilon)$ | **Mean absolute deviation (MAD)** | $\frac{1}{N_{p}}\sum_{i=1}^{N_{p}} \left\vert X\left( i \right)-\bar{X} \right\vert$ |
| **Minimum** | min($X$) | **Robust Mean Absolute Deviation**  **(rMAD)** | $\frac{1}{N_{10-90}}\sum_{i=1}^{N_{10-09}} \left\vert X_{10-90}\left( i \right)-\bar{X}_{10-90} \right\vert$ |
| **10^th^ percentile** | The 10^th^ percentile of $X$ | **Root mean squared (RMS)** | $\sqrt{\frac{1}{N_{p}}\sum_{i=1}^{N_{p}} {(X\left( i \right)+c)}^{2}}$ |
| **90^th^ percentile** | The 90^th^ percentile of $X$ | **Skewness** | $\frac{\frac{1}{N_{p}}\sum_{i=1}^{N_{p}} {(X\left( i \right)-\bar{X})}^{3}}{\left( \sqrt{\frac{1}{N_{p}}\sum_{i=1}^{N_{p}} {(X\left( i \right)-\bar{X})}^{2}} \right)^{3}}$ |
| **Maximum** | max($X$) | **Kurtosis** | $\frac{\frac{1}{N_{p}}\sum_{i=1}^{N_{p}} {(X\left( i \right)-\bar{X})}^{4}}{\left( \frac{1}{N_{p}}\sum_{i=1}^{N_{p}} {(X\left( i \right)-\bar{X})}^{2} \right)^{2}}$ |
| **Mean** | $\frac{1}{N_{p}}\sum_{i=1}^{N_{p}} X\left( i \right)$ | **Variance** | $\frac{1}{N_{p}}\sum_{i=1}^{N_{p}} {(X\left( i \right)-\bar{X})}^{2}$ |
| **Median** | The median gray-level intensity within the ROI. | **Uniformity** | $\sum_{i=1}^{N_{g}} {p(i)}^{2}$ |

***3. Second-order features***

Second-order features, widely used in pattern recognition, refer to higher order statistical measures and summarize the local spatial arrangement of intensities.

***3-1. Gray level co-occurrence matrix (GLCM) features***

Gray level co-occurrence matrix (GLCM) of size $N_{g}\times N_{g}$ described the second-order joint probability function of an image region constrained by the mask and was defined as $P(i, j|\delta, \theta)$. The ${(i, j)}^{th}$ element of this matrix represented the number of times the combination of levels $i$ and $j$ occurred in two pixels in the image, that were separated by a distance of δ pixels along angle θ. The distance δ from the center voxel was defined as the distance according to the infinity norm. For δ = 1, this resulted in 2 neighbors for each of 13 angles in 3D (26-connectivity), and a 98-connectivity for δ = 2.

Let ϵ be an arbitrarily small positive number (≈ 2.2 × 10^−16^), $P\left( i, j \right)$ be the co-occurrence matrix for an arbitrary δ and θ, $p\left( i, j \right)$ be the normalized co-occurrence matrix and equal to $\frac{P\left( i, j \right)}{\sum P\left( i, j \right)}$, $N_{g}$ be the number of discrete intensity levels in the image, $p_{x}\left( i \right)=\sum_{j=1}^{N_{g}} P\left( i, j \right)$  be the marginal row probabilities, $p_{x}\left( i \right)=\sum_{j=1}^{N_{g}} P\left( i, j \right)$ be the marginal column probabilities, $\mu_{x}$ be the mean gray level intensity of $p_{x}$and defined as $\mu_{x}=\sum_{i=1}^{N_{s}} p_{x}\left( i \right)i$, $\mu_{y}$ be the mean gray level intensity of $p_{y}$and defined as $\mu_{y}=\sum_{j=1}^{N_{s}} p_{y}\left( j \right)j$, $\sigma_{x}$ be the standard deviation of $p_{x}$, $\sigma_{y}$ be the standard deviation of $p_{y}$, $p_{x+y}\left( k \right)=\sum_{i=1}^{N_{g}} \sum_{j=1}^{N_{g}} p\left( i,j \right)$, where $i+j=k, and k=2,3,\cdots,2N_{g}$, $p_{x-y}\left( k \right)=\sum_{i=1}^{N_{g}} \sum_{j=1}^{N_{g}} p\left( i,j \right)$, where $\left| i-j \right|=k,$and $k=0,1,\cdots,N_{g}-1$, HX = $-\sum_{i=1}^{N_{g}} p_{x}(i)\log_{2} \left( p_{x}\left( i \right)+\epsilon\right)$ be the entropy of $p_{x}$,

HY = $-\sum_{j=1}^{N_{g}} p_{y}(j)\log_{2} \left( p_{y}\left( j \right)+\epsilon\right)$ be the entropy of $p_{y}$,

HXY = $-\sum_{i=1}^{N_{g}} \sum_{j=1}^{N_{g}} p\left( i,j \right)\log_{2} \left( p\left( i,j \right)+\epsilon\right)$ be the entropy of $p\left( i,j \right),$

HXY1 = $-\sum_{i=1}^{N_{g}} \sum_{j=1}^{N_{g}} p\left( i,j \right)\log_{2} \left( {p_{x}\left( i \right)p}_{y}\left( j \right)+\epsilon\right)$, and

HXY2 = $-\sum_{i=1}^{N_{g}} \sum_{j=1}^{N_{g}} {p_{x}\left( i \right)p}_{y}\left( j \right)\log_{2} \left( {p_{x}\left( i \right)p}_{y}\left( j \right)+\epsilon\right)$.

| **Autocorrelation** | $\sum_{i=1}^{N_{g}} \sum_{j=1}^{N_{g}} p\left( i,j \right)ij$ | **Joint average** | $\sum_{i=1}^{N_{g}} \sum_{j=1}^{N_{g}} p\left( i,j \right)i$ |
| --- | --- | --- | --- |
| **Cluster prominence** | $\sum_{i=1}^{N_{g}} \sum_{j=1}^{N_{g}} \left( i+j-\mu_{x}-\mu_{y} \right)^{4}p\left( i,j \right)$ | **Inverse difference (ID)** | $\sum_{k=0}^{N_{g}-1} \frac{p_{x-y}\left( k \right)}{1+k}$ |
| **Cluster shade** | $\sum_{i=1}^{N_{g}} \sum_{j=1}^{N_{g}} \left( i+j-\mu_{x}-\mu_{y} \right)^{3}p\left( i,j \right)$ | **Inverse difference normalized (IDN)** | $\sum_{k=0}^{N_{g}-1} \frac{p_{x-y}\left( k \right)}{1+\left( \frac{k}{N_{g}} \right)}$ |
| **Cluster tendency** | $\sum_{i=1}^{N_{g}} \sum_{j=1}^{N_{g}} \left( i+j-\mu_{x}-\mu_{y} \right)^{2}p\left( i,j \right)$ | **Inverse difference moment (IDM)** | $\sum_{k=0}^{N_{g}-1} \frac{p_{x-y}\left( k \right)}{1+k^{2}}$ |
| **Contrast** | $\sum_{i=1}^{N_{g}} \sum_{j=1}^{N_{g}} \left( i-j \right)^{2}p\left( i,j \right)$ | **Inverse difference moment normalized (IDMN)** | $\sum_{k=0}^{N_{g}-1} \frac{p_{x-y}\left( k \right)}{1+\left( \frac{k^{2}}{{N_{g}}^{2}} \right)}$ |
| **Correlation** | $\frac{\sum_{i=1}^{N_{g}} \sum_{j=1}^{N_{g}} p\left( i,j \right)ij-\mu_{x}\mu_{y}}{\sigma_{x}\left( i \right)\sigma_{y}(i)}$ | **Inverse variance** | $\sum_{k=1}^{N_{g}-1} \frac{p_{x-y}\left( k \right)}{k^{2}}$ |
| **Difference average** | $\sum_{k=0}^{N_{g}-1} kp_{x-y}\left( k \right)$ | **Joint energy** | $\sum_{i=1}^{N_{g}} \sum_{j=1}^{N_{g}} \left( p(i,j) \right)^{2}$ |
| **Difference entropy** | $\sum_{k=0}^{N_{g}-1} {p_{x-y}\left( k \right)log}_{2}\left( p_{x-y}\left( k \right)+\epsilon\right)$ | **Joint entropy** | $-\sum_{i=1}^{N_{g}} \sum_{j=1}^{N_{g}} p(i,j)\log_{2} \left( p(i,j)+\epsilon\right)$ |
| **Difference variance** | $\sum_{k=0}^{N_{g}-1} \left( k-DA \right)^{2}p_{x-y}\left( k \right)$ | **Maximum probability** | $max(p\left( i,j \right))$ |
| **Sum of squares** | $\sum_{i=1}^{N_{g}} \sum_{j=1}^{N_{g}} \left( i-\mu_{x} \right)^{2}p(i,j)$ | **Maximal correlation coefficient (MCC)** | $\sqrt{\begin{aligned} second largest \\ eigenvalue of Q \end{aligned}}$  $Q\left( i,j \right)=\sum_{k=0}^{N_{g}} \frac{p\left( i,k \right)p(j,k)}{p_{x}(i)p_{y}(k)}$ |
| **Sum average** | $\sum_{k=2}^{2N_{g}} p_{x+y}\left( k \right)k$ | **Informational measure of correlation (IMC) 1** | $\frac{HXY-HXY1}{max\left\{ HX,HY \right\}}$ |
| **Sum entropy** | $\sum_{k=2}^{2N_{g}} p_{x+y}\left( k \right)\log_{2} p_{x+y}\left( k \right)+\epsilon)$ | **Informational measure of correlation (IMC) 2** | $\sqrt{1-e^{-2(HXY2-HXY)}}$ |

***3-2. Gray level run length matrix (GLRLM) features***

Gray level run length matrix (GLRLM) quantifies gray level runs, which are defined as the length in number of pixels, of consecutive pixels that had the same gray level value. In a gray level run length matrix $P(i,j|\theta)$, the ${(i,j)}^{th}$element described the number of runs with gray level $i$ and length $i$ occurred in the image (ROI) along angle $\theta$.

Let $N_{g}$ be the number of discreet intensity values in the image, $N_{s}$ be the number of discreet zone sizes in the image, $N_{r}$ be the number of discreet run lengths in the image, $N_{p}$ be the number of voxels in the image,$N_{r}\left( \theta\right)$ be the number of runs in the image along angle $\theta$, which is equal to $\sum_{i=1}^{N_{g}} \sum_{j=1}^{N_{g}} P(i,j|\theta)$ and 1$\leq N_{r}\left( \theta\right)\leq N_{p}$, $P\left( i,j|\theta\right)$ be the run length matrix for an arbitrary direction $\theta$, and $p(i,j|\theta)$ be the normalized run length matrix, defined as $p\left( i,j|\theta\right)=\frac{P(i,j|\theta)}{N_{r}\left( \theta\right)}$.

| **Short run emphasis (SRE)** | $\frac{\sum_{i=1}^{N_{g}} \sum_{j=1}^{N_{r}} \frac{P(i,j\vert\theta)}{j^{2}}}{N_{r}\left( \theta\right)}$ | **Run variance (RV)** | $\sum_{i=1}^{N_{g}} \sum_{j=1}^{N_{r}} {p(i,j\vert\theta)\left( j-\mu\right)}^{2}$  $\mu=\sum_{i=1}^{N_{g}} \sum_{j=1}^{N_{r}} p\left( i,j\vert\theta\right)j$ |
| --- | --- | --- | --- |
| **Long run emphasis (LRE)** | $\frac{\sum_{i=1}^{N_{g}} \sum_{j=1}^{N_{r}} P(i,j\vert\theta)j^{2}}{N_{r}\left( \theta\right)}$ | **Run entropy (RE)** | $-\sum_{i=1}^{N_{g}} \sum_{j=1}^{N_{r}} p\left( i,j\vert\theta\right)\log_{2}$  $\left( p\left( i,j\vert\theta\right)+ \epsilon\right)$ |
| **Gray level non-uniformity (GLN)** | $\frac{\sum_{i=1}^{N_{g}} \left( \sum_{j=1}^{N_{r}} P(i,j\vert\theta) \right)^{2}}{N_{r}\left( \theta\right)}$ | **Low gray level run emphasis (LGLRE)** | $\frac{\sum_{i=1}^{N_{g}} \sum_{j=1}^{N_{r}} \frac{P(i,j\vert\theta)}{i^{2}}}{N_{r}\left( \theta\right)}$ |
| **Gray level non-uniformity normalized (GLNN)** | $\frac{\sum_{i=1}^{N_{g}} \left( \sum_{j=1}^{N_{r}} P(i,j\vert\theta) \right)^{2}}{{N_{r}\left( \theta\right)}^{2}}$ | **High gray level run emphasis (HGLRE)** | $\frac{\sum_{i=1}^{N_{g}} \sum_{j=1}^{N_{r}} P(i,j\vert\theta)i^{2}}{N_{r}\left( \theta\right)}$ |
| **Run length non-uniformity (RLN)** | $\frac{\sum_{j=1}^{N_{r}} \left( \sum_{i=1}^{N_{g}} P(i,j\vert\theta) \right)^{2}}{N_{r}\left( \theta\right)}$ | **Short run low gray level emphasis (SRLGLE)** | $\frac{\sum_{i=1}^{N_{g}} \sum_{j=1}^{N_{r}} \frac{P(i,j\vert\theta)}{i^{2}j^{2}}}{N_{r}\left( \theta\right)}$ |
| **Run length non-uniformity normalized (RLNN)** | $\frac{\sum_{j=1}^{N_{r}} \left( \sum_{i=1}^{N_{g}} P(i,j\vert\theta) \right)^{2}}{{N_{r}\left( \theta\right)}^{2}}$ | **Short run high gray level emphasis (SRHGLE)** | $\frac{\sum_{i=1}^{N_{g}} \sum_{j=1}^{N_{r}} \frac{P(i,j\vert\theta)i^{2}}{j^{2}}}{N_{z}}$ |
| **Run percentage (RP)** | $\frac{N_{r}\left( \theta\right)}{N_{p}}$ | **Long run low gray level emphasis (LRLGRE)** | $\frac{\sum_{i=1}^{N_{g}} \sum_{j=1}^{N_{r}} \frac{P(i,j\vert\theta)j^{2}}{i^{2}}}{N_{r}\left( \theta\right)}$ |
| **Gray level variance (GLV)** | $\sum_{i=1}^{N_{g}} \sum_{j=1}^{N_{r}} {p\left( i,j\vert\theta\right)\left( i-\mu\right)}^{2}$  $\mu=\sum_{i=1}^{N_{g}} \sum_{j=1}^{N_{r}} p\left( i,j\vert\theta\right)i$ | **Long run high gray level emphasis (LRHGLE)** | $\frac{\sum_{i=1}^{N_{g}} \sum_{j=1}^{N_{r}} P(i,j\vert\theta)i^{2}j^{2}}{N_{r}\left( \theta\right)}$ |

***3-3. Gray level size zone matrix (GLSZM) features***

Gray level size zone matrix (GLSZM) quantifies gray level zones in an image. A gray level zone was defined as the number of connected voxels that shared the same gray level intensity. A voxel was considered connected if the distance was 1 according to the infinity norm (26-connected region in a 3D and 8-connected region in 2D). In a gray level size zone matrix $p(i,j)$, the ${(i,j)}^{th}$ element equaled the number of zones with gray level $i$ and size $j$ appeared in image. Contrary to the GLCM and GLRLM, the GLSZM was rotation independent, with only one matrix calculated for all directions in the ROI.

Let $N_{g}$ be the number of discreet intensity values in the image, $N_{s}$ be the number of discreet zone sizes in the image, $N_{p}$ be the number of voxels in the image, $N_{z}$ be the number of zones in the ROI, which is equal to $\sum_{i=1}^{N_{g}} \sum_{j=1}^{N_{g}} P(i,j)$ and 1$\leq N_{z}\leq N_{p}$, $P\left( i,j \right)$ be the size zone matrix, and $p(i,j)$ be the normalized size zone matrix, defined as $p\left( i,j \right)=\frac{P(i,j)}{N_{z}}$.

| **Small area emphasis (SAE)** | $\frac{\sum_{i=1}^{N_{g}} \sum_{j=1}^{N_{s}} \frac{P(i,j)}{j^{2}}}{N_{z}}$ | **Zone variance (ZV)** | $\sum_{i=1}^{N_{g}} \sum_{j=1}^{N_{g}} {p(i,j)\left( j-\mu\right)}^{2}$  $\mu=\sum_{i=1}^{N_{g}} \sum_{j=1}^{N_{g}} p\left( i,j \right)j$ |
| --- | --- | --- | --- |
| **Large area emphasis (LAE)** | $\frac{\sum_{i=1}^{N_{g}} \sum_{j=1}^{N_{s}} P(i,j)j^{2}}{N_{z}}$ | **Zone entropy (ZE)** | $-\sum_{i=1}^{N_{g}} \sum_{j=1}^{N_{s}} p\left( i,j \right)\log_{2}$  $( p\left( i,j \right)+\epsilon)$ |
| **Gray level non-uniformity (GLN)** | $\frac{\sum_{i=1}^{N_{g}} \left( \sum_{j=1}^{N_{s}} P(i,j) \right)^{2}}{N_{z}}$ | **Low gray level zone emphasis (LGLZE)** | $\frac{\sum_{i=1}^{N_{g}} \sum_{j=1}^{N_{s}} \frac{P(i,j)}{i^{2}}}{N_{z}}$ |
| **Gray level non-uniformity normalized (GLNN)** | $\frac{\sum_{i=1}^{N_{g}} \left( \sum_{j=1}^{N_{s}} P(i,j) \right)^{2}}{{N_{z}}^{2}}$ | **High gray level zone emphasis (HGLZE)** | $\frac{\sum_{i=1}^{N_{g}} \sum_{j=1}^{N_{s}} P(i,j)i^{2}}{N_{z}}$ |
| **Size-zone non-uniformity (SZN)** | $\frac{\sum_{j=1}^{N_{s}} \left( \sum_{i=1}^{N_{g}} P(i,j) \right)^{2}}{N_{z}}$ | **Small area low gray level emphasis (SALGLE)** | $\frac{\sum_{i=1}^{N_{g}} \sum_{j=1}^{N_{s}} \frac{P(i,j)}{i^{2}j^{2}}}{N_{z}}$ |
| **Size-zone non-uniformity normalized (SZNN)** | $\frac{\sum_{j=1}^{N_{s}} \left( \sum_{i=1}^{N_{g}} P(i,j) \right)^{2}}{{N_{z}}^{2}}$ | **Small area high gray level emphasis (SAHGLE)** | $\frac{\sum_{i=1}^{N_{g}} \sum_{j=1}^{N_{s}} \frac{P(i,j)i^{2}}{j^{2}}}{N_{z}}$ |
| **Zone percentage (ZP)** | $\frac{N_{z}}{N_{p}}$ | **Large area low gray level emphasis (LALGLE)** | $\frac{\sum_{i=1}^{N_{g}} \sum_{j=1}^{N_{s}} \frac{P(i,j)j^{2}}{i^{2}}}{N_{z}}$ |
| **Gray level variance (GLV)** | $\sum_{i=1}^{N_{g}} \sum_{j=1}^{N_{s}} {p\left( i,j \right)\left( i-\mu\right)}^{2}$  $\mu=\sum_{i=1}^{N_{g}} \sum_{j=1}^{N_{s}} p\left( i,j \right)i$ | **Large area high gray level emphasis (LAHGLE)** | $\frac{\sum_{i=1}^{N_{g}} \sum_{j=1}^{N_{s}} P(i,j)i^{2}j^{2}}{N_{z}}$ |

***3-4. Gray level dependence matrix (GLDM) features***

Gray level dependence matrix (GLDM) quantifies gray level dependencies in an image. A gray level dependency was defined as the number of connected voxels within distance δ that are dependent on the center voxel. A neighboring voxel with gray level $j$ was considered dependent on center voxel with gray level $i$ if |$i-j$|≤α. In a gray level dependence matrix $P(i,j)$, the  ${(i,j)}^{th}$ element described the number of times a voxel with gray level $i$ with $j$ dependent voxels in its neighborhood appears in image.

Let $N_{g}$ be the number of discreet intensity values in the image, $N_{d}$ be the number of discreet dependency sizes in the image, $N_{z}$ be the number of dependency zones in the image, which is equal to $\sum_{i=1}^{N_{g}} \sum_{j=1}^{N_{d}} P(i,j)$, $P\left( i,j \right)$ be the dependence matrix, and $p(i,j)$ be the normalized dependence matrix, defined as $p\left( i,j \right)=\frac{P(i,j)}{N_{z}}$.

| **Small dependence emphasis (SDE)** | $\frac{\sum_{i=1}^{N_{g}} \sum_{j=1}^{N_{d}} \frac{P(i,j)}{i^{2}}}{N_{z}}$ | **Dependence variance (DV)** | $\sum_{i=1}^{N_{g}} \sum_{j=1}^{N_{d}} {p(i,j)\left( j-\mu\right)}^{2}$  $\mu=\sum_{i=1}^{N_{g}} \sum_{j=1}^{N_{d}} p\left( i,j \right)j$ |
| --- | --- | --- | --- |
| **Large dependence emphasis (LDE)** | $\frac{\sum_{i=1}^{N_{g}} \sum_{j=1}^{N_{d}} P(i,j)j^{2}}{N_{z}}$ | **Low gray level emphasis (LGLE)** | $\frac{\sum_{i=1}^{N_{g}} \sum_{j=1}^{N_{d}} \frac{P(i,j)}{i^{2}}}{N_{z}}$ |
| **Gray level non-uniformity (GLN)** | $\frac{\sum_{i=1}^{N_{g}} \left( \sum_{j=1}^{N_{d}} P(i,j) \right)^{2}}{N_{z}}$ | **High gray level emphasis (HGLE)** | $\frac{\sum_{i=1}^{N_{g}} \sum_{j=1}^{N_{d}} P(i,j)i^{2}}{N_{z}}$ |
| **Dependence non-uniformity (DN)** | $\frac{\sum_{j=1}^{N_{d}} \left( \sum_{i=1}^{N_{g}} P(i,j) \right)^{2}}{N_{z}}$ | **Small dependence low gray level emphasis (SDLGLE)** | $\frac{\sum_{i=1}^{N_{g}} \sum_{j=1}^{N_{d}} \frac{P(i,j)}{i^{2}j^{2}}}{N_{z}}$ |
| **Dependence non-uniformity normalized (DNN)** | $\frac{\sum_{j=1}^{N_{d}} \left( \sum_{i=1}^{N_{g}} P(i,j) \right)^{2}}{{N_{z}}^{2}}$ | **Small dependence high gray level emphasis (SDHGLE)** | The joint distribution of small dependence with higher gray-level values. |
| **Dependence entropy (DE)** | $-\sum_{i=1}^{N_{g}} \sum_{j=1}^{N_{d}} p\left( i,j \right)\log_{2}( p\left( i,j \right)+\epsilon$) | **Large dependence high gray level emphasis (LDHGLE)** | $\frac{\sum_{i=1}^{N_{g}} \sum_{j=1}^{N_{d}} P(i,j)i^{2}j^{2}}{N_{z}}$ |
| **Gray level variance (GLV)** | $\sum_{i=1}^{N_{g}} \sum_{j=1}^{N_{d}} {p\left( i,j \right)\left( i-\mu\right)}^{2}$  $\mu=\sum_{i=1}^{N_{g}} \sum_{j=1}^{N_{d}} p\left( i,j \right)i$ | **Large dependence low gray level emphasis (LDLGLE)** | $\frac{\sum_{i=1}^{N_{g}} \sum_{j=1}^{N_{d}} \frac{P(i,j)j^{2}}{i^{2}}}{N_{z}}$ |

***3-5. Neighboring gray tone difference matrix (NGTDM) features***

Neighboring gray tone difference matrix (NGTDM) quantifies the difference between a gray value and the average gray value of its neighbors within distance δ. The sum of absolute differences for gray level $i$ was stored in the matrix.

Let $X_{gl}$ be a set of segmented voxels and $x_{gl}(j_{x}$, $j_{y}, j_{z})\in X_{gl}$ be the gray level of a voxel at postion $(j_{x}$,$j_{y}, j_{z})$, then the average gray level of the neigborhood is:

$\bar{A}_{i}=\bar{A}(j_{x}$,$j_{y}, j_{z})=\frac{1}{W}\sum_{k_{x}=-\delta}^{\delta} \sum_{k_{y}=-\delta}^{\delta} \sum_{k_{z}=-\delta}^{\delta} x_{gl}(j_{x}+k_{x}, j_{y}+k_{y}, j_{z}+k_{z}),$

where $(k_{x}$,$k, k_{z})\neq(0, 0, 0)$ and $x_{gl}(j_{x}+k_{x}, j_{y}+k_{y}, j_{z}+k_{z})\in X_{gl}$. *W* is the number of voxels in the neighborhood that are also in $X_{gl}$.

Let $n_{i}$ be the number of voxels in $X_{gl}$ with gray level $i$, $N_{v,p}$ be the total number of voxels in $X_{gl}$ and equal to $\sum n_{i}$(i.e., the number of voxels with a valid region; at least 1 neighbor). $N_{v,p}\leq N_{p}$, where $N_{p}$ is the total number of voxels in the ROI, $p_{i}$ be the gray level probability and equal to $n_{i}$/$N_{v}$, $s_{i}=\left\{ \begin{matrix} \sum^{n_{i}} \left| i-\bar{A}_{i} \right| & for & n_{i}\neq0 \\ 0 & for & n_{i}=0 \end{matrix} \right.$ be the sum of absolute differences for gray level $i$, $N_{g}$ be the number of discreet gray levels, and $N_{g,p}$be the number of gray levels where $p_{i}\neq0$.

| **Coarseness** | $\frac{1}{\sum_{i=1}^{N_{g}} p_{i}s_{i}}$ | | **Complexity** | $\frac{1}{N_{v,p}}\sum_{i=1}^{N_{g}} \sum_{j=1}^{N_{g}} \left\vert i-j \right\vert\frac{p_{i}s_{i}+p_{j}s_{j}}{p_{i}{+p}_{i}}$,  where $p_{i}\neq0, p_{j}\neq0$ |
| --- | --- | --- | --- | --- |
| **Busyness** | $\frac{\sum_{i=1}^{N_{g}} p_{i}s_{i}}{\sum_{i=1}^{N_{g}} \sum_{j=1}^{N_{g}} \left\vert{ip}_{i}-jp_{j} \right\vert}$,  where $p_{i}\neq0, p_{j}\neq0$ | | **Strength** | $\frac{\sum_{i=1}^{N_{g}} \sum_{j=1}^{N_{g}} (p_{i}+p_{j}){(i-j)}^{2}}{\sum_{i=1}^{N_{g}} s_{i}}$),  where $p_{i}\neq0, p_{j}\neq0$ |
| **Contrast** | | $\left( \frac{1}{N_{g,p}(N_{g,p}-1)}\sum_{i=1}^{N_{g}} \sum_{j=1}^{N_{g}} p_{i}p_{j}{(i-j)}^{2} \right)\left( \frac{1}{N_{v,p}}\sum_{i=1}^{N_{g}} s_{i} \right)$, where $p_{i}\neq0, p_{j}\neq0$ | | |

**S3. Data augmentation using random over-sampling technique (ROSE)**

A data augmentation strategy named random over-sampling technique (ROSE) was used to deal with class imbalance in the dataset. In binary classification task, the larger the difference between the amount of data of majority and minority class is, the worse the model performs. ROSE is a smoothed bootstrap-based technique which generates artificial balanced samples from the feature space neighborhood around the minority class, and has been proven to be effective in imbalanced datasets [1].

**S4. Bayesian optimization**

Bayesian optimization provides a principled technique based on Bayes Theorem to direct a search of a global optimization problem that is efficient and effective [2]. In contrast to random or grid search, Bayesian optimization keep track of past evaluation results and builds a probabilistic model of the objective function, called the surrogate function, that is then searched efficiently with an acquisition function before candidate samples are chosen for evaluation on the real objective function [2].

The hyperparameters of the Bayesian optimization for the best-performing base radiomics model (extra-trees with ROSE oversampling) were as follows: max_depth = 2,621, max_leaf_nodes = 6,598, min_samples_leaf = 2, min_samples_split = 6, and n_estimators = 90, respectively.

**S5. Decision curve analysis (DCA)**

Decision curve analysis (DCA) calculates a clinical “net benefit” for diagnostic models in comparison to default strategies (treat-all or -none of patients) or other models [3]. Net benefit is determined by summing the benefits (true-positive results) and subtracting the harms (false-positive results), multiplied by a weighting factor based on the relative harm of an undetected disease compared with the harm of an unnecessary treatment [4]. The relative harm is calculated with the “threshold probability *p*” by the formula *p*/(1 − *p*). Threshold probability refers to a minimum probability of having a disease that patients require to justify treatment [5]. A lower threshold probability indicates that patients consider the diagnosis of a disease more important.

**S6. SHapley Additive exPlanations (SHAP)**

SHAP is a unified framework for interpreting predictions, as the only consistent and locally accurate feature attribution method based on expectations [6, 7]. SHAP values have been proposed as a unified measure of feature importance, as they assign an importance value (φi) to each feature representing the effect of including that feature in model prediction. In other words, Shapley values estimate the feature attribution for the model prediction by calculating how much the feature increases the prediction score, comparing prediction scores of the models with and without the feature.

Force plots, showing the impact and direction of the “force” of features that makes the prediction score higher or lower, were obtained. Summary plots, showing the importance values of each feature were obtained from the neural network models, and decision plots, both showing the change of model prediction scores adding-up each feature moving from the bottom to the top of the plot, were also obtained.

**Supplementary Table**

**Supplementary Table 1.** The performance of the various radiomics models in the test set

| **Classifier** | **Oversampling** | **AUC (95% CI)** | **Accuracy (%)** | **Sensitivity (%)** | **Specificity (%)** |
| --- | --- | --- | --- | --- | --- |
| **Base classifiers** | | | | | |
| AdaBoost | None | 0.65 (0.49-0.80) | 54.0 | 83.3 | 26.9 |
|  | ROSE | 0.65 (0.50-0.81) | 66.0 | 91.7 | 42.3 |
| Extra-trees | None | 0.86 (0.76-0.96) | 76.0 | 79.2 | 73.1 |
|  | ROSE | 0.87 (0.77-0.97) | 78.0 | 83.3 | 73.1 |
| GBM | None | 0.76 (0.63-0.90) | 64.0 | 83.3 | 46.2 |
|  | ROSE | 0.78 (0.64-0.91) | 66.0 | 83.3 | 50.0 |
| GPC | None | 0.76 (0.63-0.90) | 70.0 | 70.8 | 69.2 |
|  | ROSE | 0.77 (0.64-0.91) | 68.0 | 66.7 | 69.2 |
| RF | None | 0.86 (0.75-0.96) | 76.0 | 87.5 | 65.4 |
|  | ROSE | 0.84 (0.73-0.95) | 70.0 | 83.3 | 57.7 |
| SVM | None | 0.75 (0.61-0.89) | 70.0 | 70.8 | 69.2 |
|  | ROSE | 0.77 (0.63-0.90) | 70.0 | 70.8 | 69.2 |
| **Bayesian optimization of the best performing classifier** | | | | | |
| Extra-trees | ROSE | 0.89 (0.81–0.98) | 80.0 | 83.3 | 76.9 |

AUC, area under the curve; CI, confidence interval; GBM, gradient boosting machine; GPC, Gaussian process classification; RF, random forest; SVM, support vector machine; ROSE, random over-sampling examples.

**Supplementary Figures**

**Supplementary Figure 1.** Heatmap of significant radiomics features. Each column corresponds to one participant, and each row corresponds to the *z*-score normalized radiomics features. The heatmap is grouped for the training and test sets, and healthy controls (HCs; blue) versus participants with schizophrenia (red).


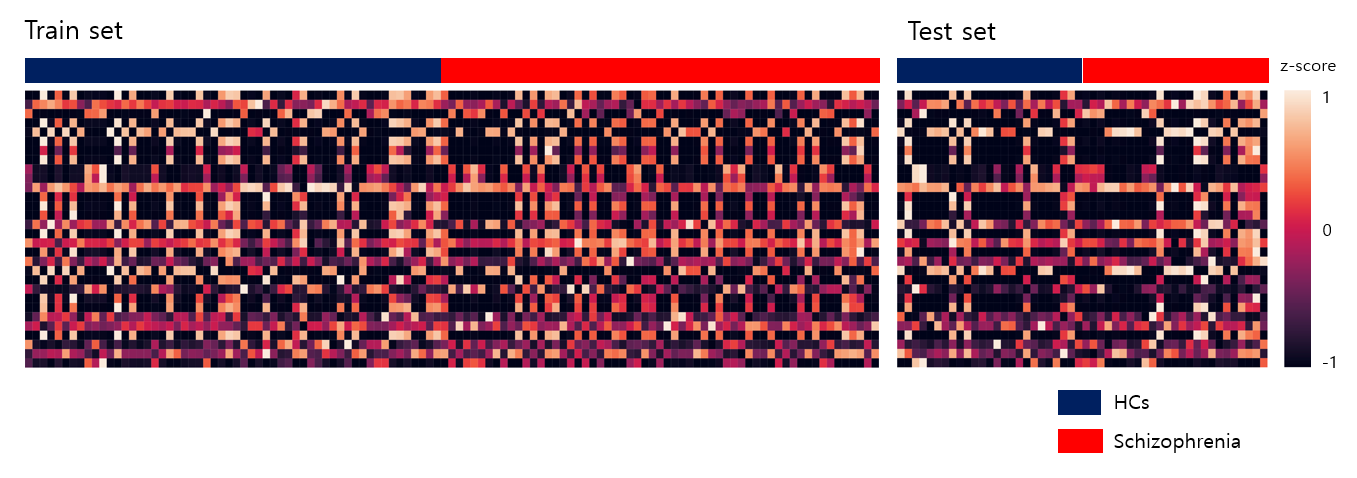


**Supplementary Figure 2.** Heatmap of AUC values achieved from the base machine learning classifiers in the (a) training set and (b) test set for differentiating participants with schizophrenia from healthy controls.


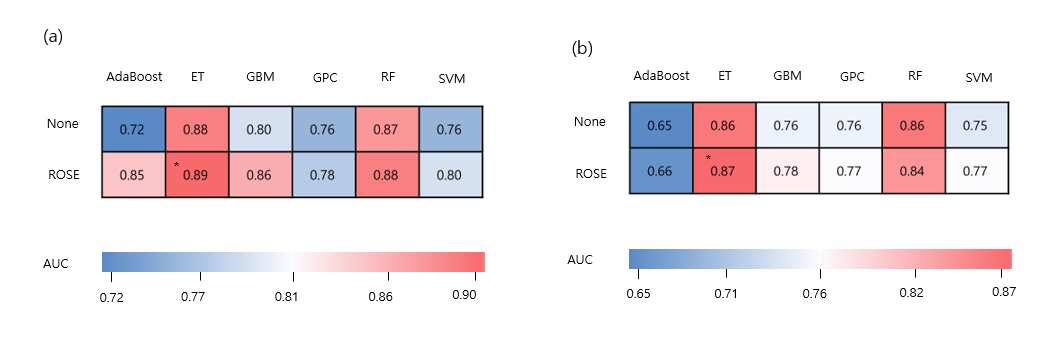


AUC, area under the curve; ET, extra-trees; GBM, gradient boosting machine; GPC, Gaussian process classification; RF, random forest; SVM, support vector machine; ROSE, random over-sampling examples.

**Supplementary Figure 3.** Representative cases of five participants (Number 1 and 2 are healthy controls [HCs] and number 3, 4, and 5 are patients with schizophrenia, respectively) with clinical application from the developed radiomics model. (A) The probability of having schizophrenia is represented in according to subject numbers. (B) The individual results of each subjects can be viewed, with interactive change of the target sensitivity. The subject number 1 (HC) was diagnosed as normal. (C) The validation results using the internal test set can be seen, and the performance can be changed according to the change of cut-off probability.


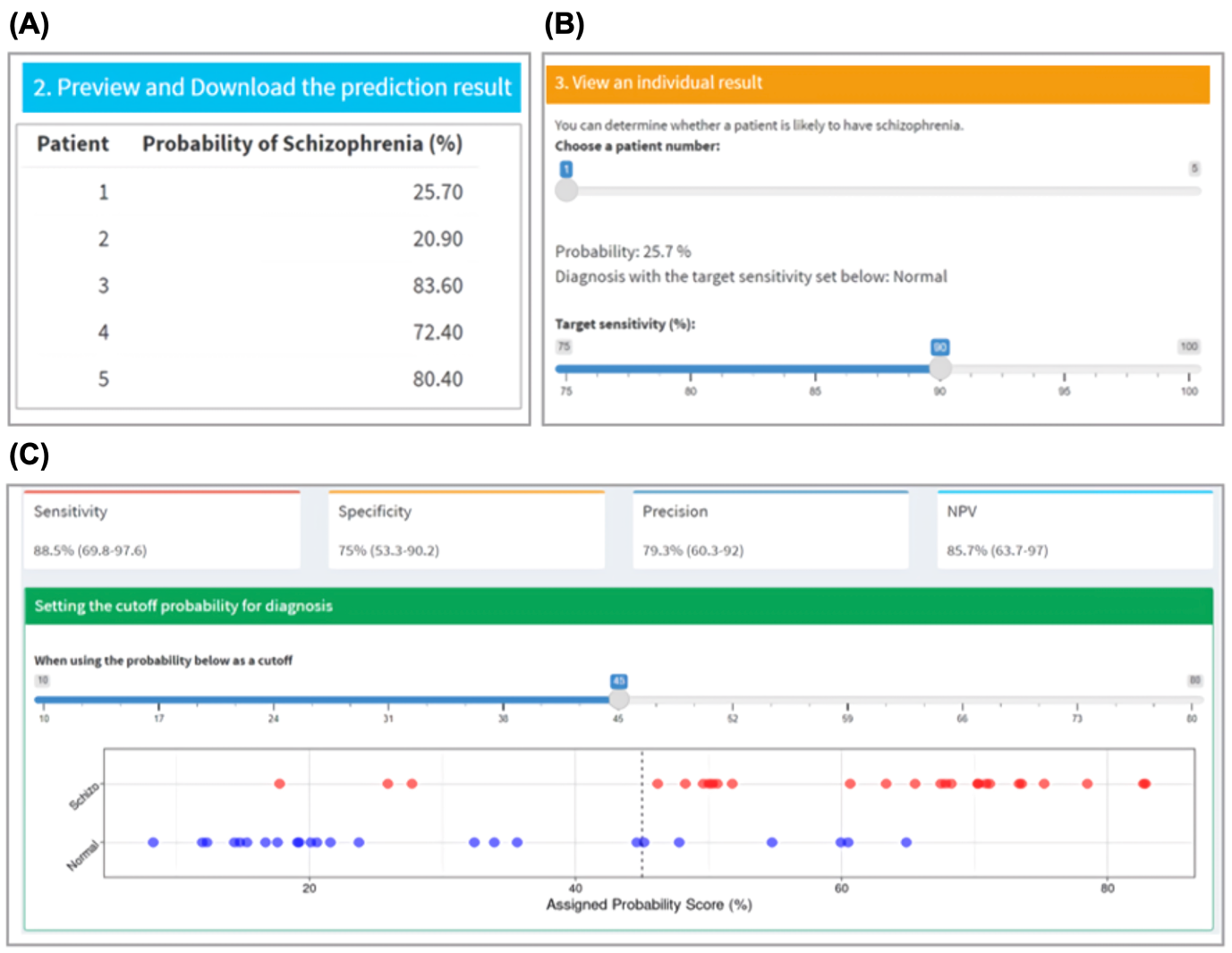


**References**

1. Lunardon N, Menardi G, Torelli N. ROSE: A Package for Binary Imbalanced Learning. The R journal 2014;6(1).

2. Snoek J, Larochelle H, Adams RP. Practical bayesian optimization of machine learning algorithms. Proceedings of the 25st international conference on neural information processing systems; 2012. p.2951–9.

3. Vickers AJ, Elkin EB. Decision curve analysis: a novel method for evaluating prediction models. Med Decis Making 2006;26(6):565-74.

4. Fitzgerald M, Saville BR, Lewis RJ. Decision curve analysis. JAMA 2015;313(4):409-10.

5. Vickers AJ, van Calster B, Steyerberg EW. A simple, step-by-step guide to interpreting decision curve analysis. Diagn Progn Res 2019;3:18.

6. Lundberg SM, Nair B, Vavilala MS, Horibe M, Eisses MJ, Adams T, et al. Explainable machine-learning predictions for the prevention of hypoxaemia during surgery. Nat Biomed Eng 2018;2(10):749-60.

7. Lundberg SM, Lee S-I. A unified approach to interpreting model predictions. Proceedings of the 31st international conference on neural information processing systems; 2017. p.4768-77.
